# Emerging biomarkers for vascular remodeling in volume and pressure overload in a cardiology cohort

**DOI:** 10.1016/j.ijcha.2025.101768

**Published:** 2025-08-07

**Authors:** Dora Csengeri, Daniel Engler, Patricia Schlieker, Amelie H. Ohlrogge, Niklas Schofer, Daniel Kalbacher, Larissa Fabritz, André Ziegler, Stefan Blankenberg, Paulus Kirchhof, Tanja Zeller, Renate B. Schnabel

**Affiliations:** a Department of Cardiology, University Heart & Vascular Center Hamburg, Hamburg, Germany; b German Center for Cardiovascular Research (DZHK), partner site Hamburg/Kiel/Luebeck, Hamburg, Germany; c University Center of Cardiovascular Science, University Heart and Vascular Center Hamburg, University Medical Center Hamburg Eppendorf, Hamburg, Germany; d Institute of Cardiovascular Sciences, University of Birmingham, Birmingham, UK; e Roche Diagnostics, Rotkreuz, Switzerland

**Keywords:** Biomarkers, Volume overload, Pressure overload, Valvular disease, Cardiac remodeling, Vascular remodeling, Endothelial dysfunction

## Abstract

Emerging evidence indicates that the circulating biomarker angiopoietin-2 (ANGPT2), bone morphogenetic protein2 10 (BMP10), fibroblast growth factor 23 (FGF23), and insulin-like growth factor-binding protein7 (IGFBP7) reflect distinct pathophysiological vascular and cardiac processes contributing to adverse cardiac remodeling in the context of volume or pressure overload. This study aims to investigate the association between these circulating biomarkers, and the presence of hemodynamically significant pressure or volume overload and adverse clinical outcomes.

**Methods:**

In an observational cohort of outpatient cardiology patients (N = 1506) the relationship between the four emerging biomarkers and pressure/volume overload using multivariable-adjusted regression models was examined.

**Results:**

Elevated levels of the four biomarkers were positively associated with the presence of significant volume overload compared to none/mild valve disease. (ANGPT2: Odds ratio (OR) 1.26 (95 % confidence interval (CI): 1.17–1.35), p < 0.001; BMP10: OR = 2.57 (95 %-CI: 1.89–3.48), p < 0.001; FGF23: OR = 1.51 (95 %-CI: 1.14–1.20), p = 0.004; IGFBP7: OR = 1.39 (95 %-CI: 1.14–1.69), p = 0.001, NT-proBNP: OR = 1.69 (95 %-CI: 1.47–1.95), p < 0.001).

FGF23 and NT-proBNP demonstrated statistically significant associations with pressure overload compared to none/mild burden.

Higher concentrations of all biomarkers were predictive of all-cause mortality in patients with volume overload.

**Conclusions:**

This study highlights that circulating biomarkers associated with distinct pathophysiological vascular pathways, including inflammation, fibrosis, and calcification, are elevated in patients with hemodynamically significant volume overload. Given their association with mortality, these biomarkers merit further investigation of their underlying pathways, particularly in conjugation with the established biomarker NT-proBNP, to clarify their potential for more targeted clinical applications.

## 1 Introduction

Valvular disease, characterizing by distinct phenotypes of volume or pressure overload, is becoming increasingly prevalent in an aging population [1]. Despite advances in treatment, there is a critical need for improved diagnostic and prognostic tools to effectively monitor progression and guide for various therapies [[2], [3], [4]]. Circulating biomarkers have emerged as promising candidates in this context, as some of them present tissue or disease specificity, may have molecular targets and often can be objectively quantified. In combination, they provide insights into the (dis)balanced underlying molecular mechanism of cardiovascular pathology and they potentially serve as predictors of clinical outcomes. Among these, angiopoietin-2 (ANGPT2), bone morphogenetic protein 10 (BMP10), fibroblast growth factor 23 (FGF23), and insulin-like growth factor-binding protein 7 (IGFBP7) have recently gained attention as indicators of vascular and cardiac dysfunction [[5], [6], [7], [8], [9]].

ANGPT2 is secreted by endothelial cells and promotes endothelial activation by inhibiting Tie2 signaling [10]. ANGPT2 is a well-established marker of endothelial activation and vascular permeability, directly linked to vascular pathophysiology and heart failure progression [[11], [12], [13]]. BMP10, known to be released specifically by the heart, is targeting vascular ALK-1 receptors and contributes to vascular quiescence [14]. BMP 10 is primarily expressed in the heart, particularly in atrial tissue, but also has been implicated in cardiac development, remodeling, and the regulation of vascular tone. In recent cardiovascular research, it has been implicated in the regulation of cardiac growth and function, with emerging evidence linking it to heart failure and hypertrophic remodeling [13,[15], [16], [17]]. While cardiac-specific, its circulating levels may reflect the interplay between cardiac and vascular adaption to hemodynamic stress, making it a candidate for exploring cardiac-vascular crosstalk. FGF23, mainly released by osteoblasts and osteocytes into the systemic circulation is acting in many organs and vessels (e.g. vascular contraction and calcification), initially being recognized for its role in mineral metabolism, it has since been identified as an independent marker of cardiovascular risk, particularly in association with left ventricular dysfunction, morbidity, and mortality [9,15,18]. IGFBP7 (“angiomodulin”), is key inflammatory cytokine released by senescent endothelial cells under stress and ageing, adversely influencing cardiomyocyte metabolism and kidney function [19]. Being a key modulator of insulin-like growth factor signaling, it has gained significance as a biomarker due to its role in promoting fibrosis, which contributes to diastolic dysfunction and heart failure [8,13,20].

These four biomarkers were selected to capture a broad spectrum of vascular and cardiac processes that may contribute to adverse remodeling – ranging from endothelial activation and cardiac-specific signaling to mineral metabolism and systemic senescence. The association of the novel biomarkers with clinical outcome has been lately shown with some cardiac disease, but their correlation with specific cardiovascular phenotypes, such as volume and pressure overload, remains to be elucidated. This study aims to investigate these experimental biomarkers and their association with adverse cardiac remodeling to improve risk stratification in patients experiencing volume or pressure overload.

## 2 Methods

### 2.1 Study design

Data from a cohort of 1506 outpatient cardiology patients enrolled between 2012 and 2023 at the University Heart and Vascular Center, Hamburg, Germany.

Patients were recruited during routine outpatient clinic visits, and data were collected from questionnaires, patient charts, and reports from external physicians. Venous blood samples were collected and immediately processed in a research laboratory. Outcomes, including heart failure, atrial fibrillation, myocardial infarction, coronary heart disease, and all-cause mortality were assessed via questionnaires, structured telephone interviews, electronic hospital, and ambulatory care records as well as death certificates. All outcomes were adjudicated by medical professionals.

### 2.2 Echocardiographic measurements

Patients were categorized based on the severity of their valvular diseases, defined by significant volume overload (moderate to severe tricuspid or mitral regurgitation), or pressure overload (moderate to severe aortic valve stenosis). Transthoracic echocardiography was performed by qualified physicians according to the American Society of Echocardiography guidelines [21]. Echocardiographic images were acquired using Philips EPIQ systems and analysed with the TomTec workstation (IMAGE-Com, TOMTEC-ARENA, Tomtec Imaging System GmbH, Unterschleissheim, Germany).

Valvular disease severity was assessed using quantitative, semiquantitative, and qualitative parameters, including ejection fraction, left ventricular chamber and annulus dimensions, tricuspid annular plane systolic excursion, effective regurgitant orifice area, regurgitant volume, vena contracta width, and colour Doppler imaging. Tricuspid and mitral regurgitation were evaluated using standard 2D colour Doppler methods and classified using a predefined scale: five classes scheme for tricuspid regurgitation (mild, moderate, severe, massive, and torrential) and four classes for mitral regurgitation (mild, moderate, moderate-to-severe, severe) [[22], [23], [24]].

### 2.3 Biomarker measurements

Venous blood samples from all patients were fractionated and stored at −80 °C until analysis. Absolute protein concentrations were centrally quantified in EDTA plasma using standardized procedures (Roche Diagnostics, Penzberg, Germany). NT-proBNP levels were measured in-house using the ELECSYS 2010 platform (ECLIA, Roche Diagnostics) via an electrochemiluminescence immunoassay with an analytical range of 5–35,000 pg/mL. The intra- and interassay coefficients of variation of 1.38 and 2.58 %, respectively.

The experimental biomarkers were measured at Roche using a cobas e601 analyzer (Roche Diagnostics, Penzberg, Germany) using noncommercial, robust prototype Elecsys electrochemiluminescence immunoassays (Roche Diagnostics, Penzberg, Germany). The coefficients of variation for Ang2 were 5.2 % and 4.0 % at concentrations of 0.98 and 3.40 ng/mL, respectively.

The BMP10 assay had a lower detection limit of 0.003  ng/mL, a functional sensitivity (lower limit of quantification) of 0.012  ng/mL and upper measuring range of 10 ng/mL. Within-run control measurements performed during the study showed a coefficient of variation of 2.35 % (mean: 1.38  ng/mL).

FGF23 levels were determined using a rabbit monoclonal antibody-based immunoassay with a functional sensitivity of 4 pg/ml and a within-run coefficient of variation of 1.7 %.

IGFBP7 concentrations were measured with a precision within-run coefficient variation of 2 % and a detection limit of 0.01 ng/ml.

All biomarkers analysis was conducted by laboratory personnel blinded to clinical data under stringent quality control and calibration protocols.

### 2.4 Statistical analysis

Continuous variables are presented as median (25th, 75th percentile) and categorical variables are presented as frequencies and percentages. For the analyses, valvular disease was classified as none/mild vs. moderate to severe. Outliers have been identified by graphical representations in boxplots and scatterplots for the biomarkers. In addition, we considered a Z-score of 2.6 or more as an outlier. Normal distribution has been analysed by the Kolmogorov-Smirnov test. We have applied natural log transformation to process the regression models.

Binary logistic regression models and Cox regression plots were used to assess the associations of these biomarkers with valvular disease severity, adjusted for age, sex, and interim valve intervention. Distribution per time point for specific parameters is assessed via boxplots. Median and 25th–75th percentile (Inter quartile range [IQR]) are shown in boxes. Cumulative incidence curves are shown based on Kaplan-Meier estimator. Log rank test is given to examine any group difference. All statistical models were conducted using SPSS 29.0 (SPSS Inc., Chicago, IL, USA). A two-tailed p-value < 0.05 was considered statistically significant and confidence intervals were set at 95 %.

The local Ethics Committee approved the observational study (PV5705). Participants signed written informed consent. The authors had full access to the data and take responsibility for its integrity. All authors have read and agreed to the manuscript as written.

## 3 Results

### 3.1 Baseline characteristics

**Fig. 1:**
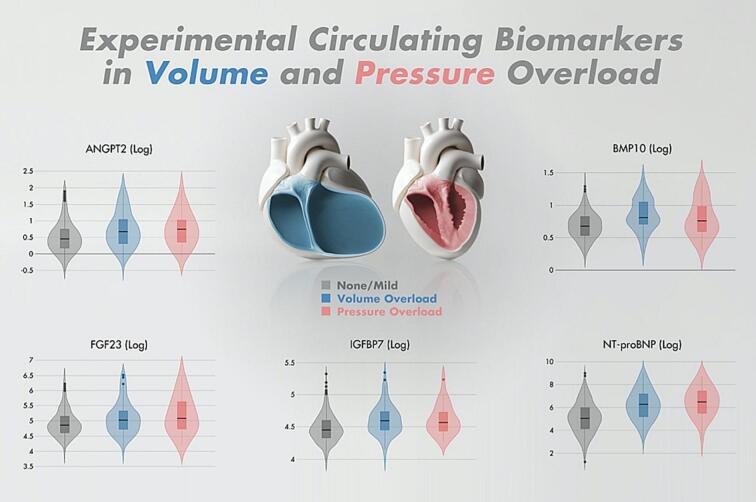
Violin plots of circulating biomarkers in patients with volume or pressure overload vs. patients without relevant valvular disease. Abbreviations: ANGPT2, angiopoietin-2; BMP10, bone morphogenetic protein 10; FGF23, fibroblast growth factor 23; IGFBP7, insulin-like growth factor-binding protein 7; NT-proBNP, N-terminal pro-B-type natriuretic peptide.

The baseline characteristics of the 1506 participants and biomarker levels of the patients are summarized in Table 1. The median age was 63 years, (IQR 51.5–71.4), and 447 participants (29.7 %) were women. Within the cohort, 170 patients (11.3 %) exhibited volume overload, and 34 patients (2.3 %) presented with pressure overload. The median follow-up was 2.6 years. The study population had a median body mass index (BMI) of 26.7 kg/m^2^, indicating that participants were slightly overweight on average. Patients with volume or pressure overload were generally older and had a higher prevalence of comorbidities compared to those without significant valvular disease. Women were more frequently represented in the group with volume overload (44.7 %). Our findings indicate that elevated levels of all circulating biomarkers were significantly associated with the presence of volume overload (Fig. 1 and Table 2). Additionally, NT-proBNP and FGF23 demonstrated a significant correlation with pressure overload, with adjusted odds ratios (OR) of 1.53 (p = 0.02) and 2.30, (p = 0.005), respectively, after adjusting for age, sex, and prior valvular interventions (Table 2).

**Table 1:** Patient characteristic by phenotypes of heart valve disease.

Variables N (%)	None/Mild valvular disease 1302 (86.5)	Volume Overload (VO) 170 (11.3)	Pressure Overload (PO) 34 (2.3)
*Risk factors*			
Age (years)	63.1 (52.2, 71.4)	69.7 (62.2, 75.2)	73.2 (66.1, 77.8)
Women, N (%)	364 (28.0)	76 (44.7)	7 (20.6)
Body mass index (kg/ m^2^)	27.1 (24.5, 30.4)	25.0 (23.0, 30.4)	28.5 (25.1, 32.2)
Smoker, N (%)	232 (17.8)	22 (12.9)	8 (23.5)

*Prior disease*			
Diabetes mellitus, N (%)	280 (21.5)	32 (18.8)	12 (35.3)
Heart failure, N (%)	232 (17.8)	60 (35.3)	15 (44.1)
Atrial fibrillation, N (%)	555 (42.6)	78 (45.9)	18 (52.9)
Dyslipidaemia, N (%)	703 (54.3)	87 (51.8)	24 (70.6)
Stroke/TIA, N (%)	234 (18.0)	21 (12.4)	11 (32.4)
Renal dysfunction, N (%)	175 (13.4)	23 (13.5)	6 (17.6)
Coronary artery disease, N (%)	638 (49.0)	87 (51.2)	28 (82.4)
Hypertension, N (%)	1029 (79.0)	143 (84.1)	27 (79.4)
Cardiac valve intervention during follow-up*, N (%)	54 (4.2)	22 (12.9)	13 (38.2)

*Biomarkers*			
NT-proBNP (pg/ml)	156 (61, 400)	538 (183, 1385)	721 (265, 2076)
ANGPT2 (ng/ml)	1.57 (1.20, 2.21)	2.10 (1.44, 3.88)	2.13 (1.43, 3.89)
BMP10 (ng/ml)	1.99 (1.72, 2.27)	2.25 (2.03, 2.89)	2.14 (1.80, 2.77)
FGF23 (pg/ml)	130 (101, 179)	164 (115, 235)	203 (125, 417)
IGFB7 (ng/ml)	86 (76, 100)	99 (85, 116)	98 (84, 122)

Coronary artery disease was defined as prior myocardial infarction or angiographic evidence of coronary artery disease. Volume overload (VO) is defined by significant mitral valve insufficiency, tricuspid valve insufficiency or both. Pressure overload (PO) is defined by significant aortic valve stenosis without presence of other valve disease. *cardiac valve interventions comprised catheter-based or surgical repair/replacement. Data are presented as percentages for categorical variables and as median (25th, 75th percentile) for continuous variables. Abbreviations: ANGPT2, angiopoietin-2; BMP10, bone morphogenetic protein10; FGF23, fibroblast growth factor 23; IGFBP7, insulin-like growth factor-binding protein 7; NT-proBNP, N-terminal pro–B-type natriuretic peptide; TIA, transitory ischemic attack.

**Table 2:** Associations between biomarker and volume or pressure overload.

Biomarker	Volume overload		Pressure overload	
	OR (95 % CI)	P value	OR (95 % CI)	P value
NT-proBNP	1.81 (1.59–2.06)	<0.001	2.13 (1.64–2.77)	<0.001
	1.69 (1.465–1.95)	<0.001	1.53 (1.07–2.20)	0.020
ANGPT 2	4.78 (2.35–9.72)	<0.001	1.16 (1.02–1.31)	0.022
	1.26 (1.17–1.35)	<0.001	1.08 (0.89–1.30)	0.44
BMP 10	3.30 (2.46–4.43)	<0.001	2.84 (1.70–4.73)	<0.001
	2.57 (1.89–3.48)	<0.001	1.26 (0.52–3.04)	0.61
FGF 23	1.85 (1.44–2.39	<0.001	1.49 (0.89–2.51)	0.13
	1.51 (1.14–2.00)	0.004	2.30 (1.29–4.11)	0.005
IGFBP 7	1.66 (1.41–1.96)	<0.001	1.51 (1.01–2.26)	0.043
	1.39 (1.14–1.69)	0.001	0.78 (0.47–1.29)	0.33

Provided are odd ratios for log-transformed biomarkers, 95% confidence intervals and p values. Abbreviation: ANGPT2, angiopoietin-2; BMP10, bone morphogenetic protein 10; CI, confidence interval; FGF23, fibroblast growth factor 23; IGFBP7, insulin-like growth factor-binding protein 7; NT-proBNP, N-terminal pro–B-type natriuretic peptide; OR, odds ratio.

Upper row: crude odds ratios, lower row OR adjusted for age, sex and interim valvular interventions.

**Fig. 2:**
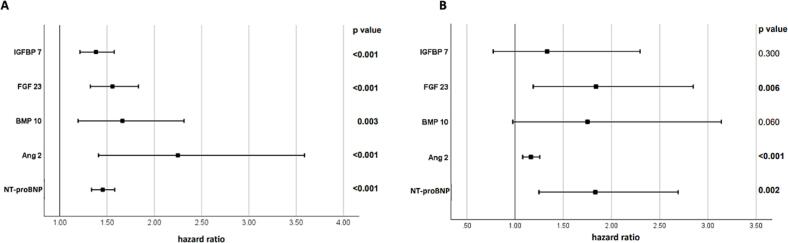
Cox-regression models adjusted for age, sex and interim valvular interventions. A: for the combined outcome of incident heart failure, atrial fibrillation, myocardial infarction, coronary heart disease and all-cause mortality and B: for all-cause mortality in patients with volume overload during 5 years of follow-up. Displayed are hazard ratios on log-transformed biomarkers, 95 % confidence intervals and p values.

Elevated levels of all circulating biomarkers were associated with the combined outcome in patients with volume overload. Specifically, log-transformed ANGPT2 was associated with a hazard ratio of 1.165, (p < 0.001), log-transformed FGF23 with an HR of 1.84 (p-value 0.006), and log-transformed NT-proBNP were statistically significantly related to all-cause death (Fig. 2).

**Fig. 3:**
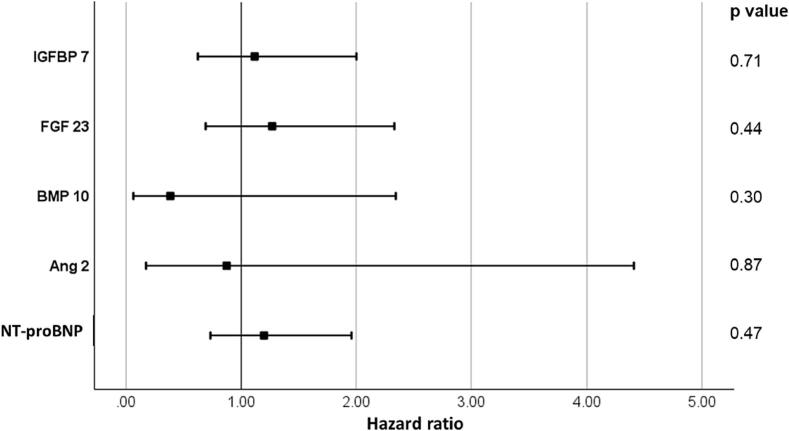
Cox-regression model adjusted for age, sex and interim valvular interventions for the combined outcome of incident heart failure, atrial fibrillation, coronary heart disease (defined as prior myocardial infarction or angiographic evidence of coronary artery disease) and all-cause mortality in patients with pressure overload during 5 years of follow-up. Displayed are hazard ratios on log-transformed biomarkers, 95 % confidence intervals and p values.

For patients with pressure overload, no statistically significant associations were observed between circulating biomarkers and the combined outcome after adjustment for age, sex and valvular interventions (Fig. 3).

Over a median follow-up of five years, 25 participants (21.6 %) with volume overload and 10 participants (35.5 %) with pressure overload died. After multivariable adjustment, elevated BMP10 levels showed a borderline association with increased all-cause mortality in patients with volume overload patients (HR = 1.64; p = 0.002; Table 3). Higher concentrations of the other four biomarkers demonstrated predictive value for mortality in patients with volume-overloaded.

**Table 3:** Outcomes in patients with volume overload vs. none/mild valve disease.

Biomarker	All-cause mortality		Combined outcomes	
	Hazard ratio (95 % CI)	P value	Hazard ratio (95 % CI)	P value
NT-proBNP	1.72 (1.47–1.995)	<0.001	1.32 (1.21–1.43)	<0.001
ANGPT 2	3.72 (1.67–8.325)	0.001	2.76 (1.79–4.25)	<0.001
BMP 10	1.64 (1.195–2.26)	0.002	1.58 (1.31–1.91)	<0.001
FGF 23	2.08 (1.65–2.62)	<0.001	1.48 (1.26–1.73	<0.001
IGFBP 7	1.55 (1.25–1.91)	<0.001	1.21 (1.07–1.37)	0.002

All-cause mortality and combined endpoints in patients with volume overload vs. none/mild valve disease adjusted for age, sex and interim valvular interventions. Combined outcome includes incident heart failure, atrial fibrillation, myocardial infarction, coronary heart disease and all-cause mortality. Provided are hazard ratios, 95% confidence intervals and p values for log-transformed biomarkers. Abbreviation: ANGPT2, angiopoietin-2; BMP10, bone morphogenetic protein 10; CI, confidence interval; FGF23, fibroblast growth factor 23; IGFBP7, insulin-like growth factor-binding protein 7; NT-proBNP, N-terminal pro–B-type natriuretic peptide; HR, hazard ratio.

## 4 Discussion

This study highlights the association between the emerging circulating biomarkers of vascular and cardiac remodeling ANGPT2, BMP10, FGF23, and IGFBP7 with hemodynamically significant volume and pressure overload. While all biomarkers were significantly related to cardiovascular outcomes and all-cause mortality in patients with volume overload, no statistically significant associations were observed for outcomes in patients with pressure overload after multivariable-adjustment, suggesting different response to vascular stress and tissue adaptation.

### 4.1 Volume overload

Volume overload leads to distinct pathophysiological processes, including cardiac dysfunction and remodeling. Biomarkers capture these dynamic processes, offering a critical tool for assessing disease progression [16,20,[25], [26], [27]]. The observed associations between these biomarkers, significant volume overload and increased mortality are clinically meaningful. NT-proBNP, a well-established marker for diagnosing heart failure and predicting cardiovascular outcomes serves as a reliable benchmark for increased myocyte stretch and myocardial wall tension. The findings of this study suggest that emerging biomarkers of vascular and cardiac remodeling provide a more nuanced understanding of cardiovascular pathology: the median concentrations of BMP10 and IGFBP7 were lower in pressure overload as compared to volume overload, which is different for NT-proBNP and Angp2.

Elevated levels of ANGPT2, BMP10, IGFBP7, and FGF23 were strongly associated with the presence of volume overload. Prior studies have shown that high ANGPT2 levels are linked to poor outcomes, including increased risks of hospitalization and mortality in heart failure patients [11,26]. Similarly, elevated IGFBP7 levels are associated with adverse outcomes in heart failure, particularly in heart failure with preserved ejection fraction (HFpEF), aiding in diagnosis, prognosis, and prediction of hospitalization and mortality [28,29]. IGFBP7 also correlates with immune regulation, inflammation, and cardiac remodeling [13,30,31]. FGF23 has been implicated in worse clinical outcomes in HFpEF, including reduced exercise capacity, and increased risk of death or hospitalization. Its association with renal dysfunction, anemia, and myocardial remodeling underscores its multifactorial role in HFpEF pathophysiology, potentially mediated by its effects on left ventricular hypertrophy, fibrosis, and iron metabolism [13,32].

BMP10, an emerging biomarker for atrial remodeling and stress, is particularly relevant in conditions like atrial fibrillation, and mitral valve regurgitation. Its role in extracellular matrix organization, cytoskeletal dynamics, and atrial dilatation suggests utility in identifying patients at risk for or those who might benefit from interventions targeting atrial remodeling [15,[33], [34], [35]]. These findings highlight the potential clinical value of these biomarkers in monitoring and managing volume overload-related patients’ complications.

### 4.2 Pressure overload

After correction for age, sex and interim valvular interventions, FGF23 was the only biomarker next to NT-proBNP significantly associated with pressure overload, consistent with its role in ventricular hypertrophy and myocardial damage [[35], [36], [37], [38]].

The associations of the other experimental biomarkers lost statistical significance of their associations after adjustment for confounders. ANGPT2 is primarily considered a vascular and angiogenic marker, whereas BMP10 is highly cardiac-specific, especially related to atrial tissue and atrial fibrillation risk. Interestingly, both ANGPT2 and BMP10 were significantly associated with adverse outcomes in the context of volume overload, but not in pressure overload. This pattern suggests that remodeling in volume overload may involve both vascular mechanisms (e.g., endothelial activation or angiogenesis reflected by ANGPT2) and atrial/cardiac-specific processes (reflected by BMP10). In contrast, pressure overload may activate alternative or additional pathways not captured by these markers. The lack of significant associations in pressure overload could indicate differences in the type of structural adaptation or neurohormonal activation that predominates in each condition. Although lower than in heart failure, the elevated NT-proBNP levels in pressure overload (721 pg/mL) reflected the cardiovascular risk factors in the pathophysiology of increased myocyte stretch.

### 4.3 Mortality

FGF23, IGFBP 7, and ANGPT2 were significant predictors of all-cause mortality, reinforcing their potential as prognostic markers. These results align with prior research showing demonstrating their involvement in pathways leading to cardiovascular mortality [5,8,9,16,20,[25], [26], [27]]. NT-proBNP also remained a strong predictor of mortality, underscoring its importance in risk stratification for heart failure patients [39,40]. In patients with volume overload, BMP10 exhibited a borderline association with all-cause mortality, suggesting it may have a less prominent role compared to other biomarkers and/or other reasons to mortality beyond cardiac reasons.

### 4.4 Limitations and Strengths

This study has several limitations: The small sample size may have reduced the power to detect group differences, particularly in patients with pressure overload. Variability in biomarker levels due to comorbidities, differences in disease severity, and the timing of sample collection could all contribute to inconsistencies. Larger, more diverse cohorts are needed to validate these findings.

Strengths of this study include the use of a well-characterized cohort with clearly defined volume and pressure overload phenotypes that capture diverse aspects of cardiac pathophysiology.

## 5 Conclusion

This study highlights the potential benefit of adding emerging biomarkers such as ANGPT2, BMP10, FGF23, and IGFBP7 to traditional markers like NT-proBNP [8,9,16,20,26,38,41,42]. These markers capture different aspects of vascular inflammation, fibrosis, and remodeling, providing a more detailed picture of volume overload. Their use could improve risk stratification, enable earlier interventions, and guide targeted therapies, ultimately enhancing outcomes for patients with hemodynamically significant volume overload. While NT-proBNP remains essential, combining it with these biomarkers may refine vascular risk assessment and warrants further investigation.

## CRediT authorship contribution statement

**Dora Csengeri:** Writing – review & editing, Writing – original draft, Visualization, Validation, Supervision, Software, Resources, Project administration, Methodology, Investigation, Funding acquisition, Formal analysis, Data curation, Conceptualization. **Daniel Engler:** Writing – review & editing, Writing – original draft, Visualization, Validation, Supervision, Software, Resources, Project administration, Methodology, Investigation, Funding acquisition, Formal analysis, Data curation, Conceptualization. **Patricia Schlieker:** Software, Project administration, Methodology, Formal analysis, Data curation. **Amelie H. Ohlrogge:** Writing – review & editing, Writing – original draft, Validation, Methodology, Data curation. **Niklas Schofer:** Writing – review & editing, Writing – original draft, Supervision, Project administration, Funding acquisition. **Daniel Kalbacher:** Writing – review & editing, Visualization, Supervision, Data curation. **Larissa Fabritz:** Writing – review & editing, Writing – original draft, Validation, Supervision, Resources, Funding acquisition, Data curation. **André Ziegler:** Writing – review & editing, Writing – original draft, Validation, Supervision, Resources, Methodology, Data curation. **Stefan Blankenberg:** Writing – review & editing, Writing – original draft, Supervision, Resources, Funding acquisition, Data curation. **Paulus Kirchhof:** Writing – review & editing, Writing – original draft, Methodology, Funding acquisition. **Tanja Zeller:** Writing – review & editing, Resources, Methodology, Funding acquisition, Formal analysis, Data curation. **Renate B. Schnabel:** Writing – review & editing, Writing – original draft, Visualization, Validation, Supervision, Software, Resources, Project administration, Methodology, Investigation, Funding acquisition, Formal analysis, Data curation, Conceptualization.

## Funding

**LF** acknowledges support by EU grant agreement 633196 [CATCH ME], grant agreement 847770 [AFFECT-AF] and EU grant agreement 965286 [MAESTRIA], 10.13039/501100000274British Heart Foundation (AA/18/2/34218), German Center for Cardiovascular Research supported by the German Ministry of Education and Research (DZHK) and NIHR.

**PK** has been partially supported by European Union BigData@Heart (grant agreement EU IMI 116074), AFFECT-AF (grant agreement 847770), and MAESTRIA (grant agreement 965286), British Heart Foundation (PG/17/30/32961 and PG/20/22/35093; AA/18/2/34218), German Centre for Cardiovascular Research supported by the German Ministry of Education and Research (DZHK), and Leducq Foundation.

**RBS** has received funding from the European Research Council (ERC) under the European Union’s Horizon 2020 research and innovation programme under the grant agreement No 648131, from the European Union’s Horizon 2020 research and innovation programme under the grant agreement No 847770 (AFFECT-EU) and German Center for Cardiovascular Research (DZHK e.V.) (81Z1710103 and 81Z0710114); German Ministry of Research and Education (BMBF 01ZX1408A) and ERACoSysMed3 (031L0239). R.B.S has received lecture fees and advisory board fees from BMS/Pfizer and Bayer outside this work.

## Declaration of competing interest

The authors declare the following financial interests/personal relationships which may be considered as potential competing interests: **DC** received a research grant from Wolfgang Seefried Project funding of the German Heart Foundation e.V.

**DE** has no conflict of interest.

**PS** has no conflict of interest.

**AHO** has no conflict of interest.

**NS** received travel support from Edwards Lifesciences and St. Jude Medical, speaker honoria and travel support from Boston Scientific.

**DK** has received personal fees from Abbott Medical, Edwards Lifesciences, Pi-Cardia Ltd., and Medtronic Inc.

**LF** received institutional research grants by European Union, British Heart Foundation, Medical Research Council (UK), German Centre for Cardiovascular Research, and several biomedical companies active in the field of research. L.F. is listed as inventor on two issued patents held the University of Birmingham (Atrial Fibrillation Therapy WO 2015140571, Markers for Atrial Fibrillation WO 2016012783).

**AZ** is an employee of Roche Diagnostics Intl.

**SB** is supported by the Innovative medicine initiative (IMI), the Foundation Leducq, Siemens, Bayer, Astra Zeneca, Deutsche Gesetzliche Unfallversicherung (DGUV) and Novartis.

**PK** has received research support for basic, translational, and clinical research projects from European Union, British Heart Foundation, Leducq Foundation, Medical Research Council (UK), German Centre for Cardiovascular Research, and from several drug and device companies active in atrial fibrillation; is listed on 2 patents held by the University of Birmingham (Atrial Fibrillation Therapy WO 2015140571 and Markers for Atrial Fibrillation WO 2016012783); and has been partially supported by European Union BigData@Heart, AFFECT-AF, and MAESTRIA, British Heart Foundation, German Centre for Cardiovascular Research supported by the German Ministry of Education and Research (DZHK), and Leducq Foundation.

**TZ** has received funding from the German Research Foundation, the EU Horizon 2020 programme, the EU ERANet and ERAPreMed Programmes, the German Centre for Cardiovascular Research, the German Ministry of Education and Research, and the German Reseach foundation. TZ is listed as co-inventor of an international patent on the use of a computing device to estimate the probability of myocardial infarction (International Publication Number WO2022043229A1). TZ is a shareholder of the ART.EMIS GmbH Hamburg.

**RBS** has received funding from the European Research Council (ERC) under the European Union’s Horizon 2020 research and innovation programme, from the European Union’s Horizon 2020 research and innovation programme, and German Center for Cardiovascular Research; German Ministry of Research and Education, and ERACoSysMed3. R.B.S has received lecture fees and advisory board fees from BMS/Pfizer and Bayer outside this work.
